# Treatment of Puerperal Sepsis

**Published:** 1903-12

**Authors:** 


### ABSTRACTS.

### Treatment of Puerperal Sepsis.

SEVERAL papers have been published recently on this subject and we present three of them in abstract.

Prof. H. Fehling, of Strassburg University, in a paper on The prophylaxis and treatment of puerperal fever, *Muenchener Med. Wochenschrift,* No. 33, August 18, 1903, says:

That operative procedure in this infection being of comparatively little use, the search for systemic remedies to neutralise the general poisoning has been unremitting. There can be no doubt, he continues, that collargolum, intravenously administered, is of value. Sterile 2 per cent. solutions were employed, 2½ to 5 drams thereof being injected with a fine needle into a vein at the elbow-bend. With a little practice this is easily done. There was never any ill effect. The results varied; some charts showed an immediate fall in pulse and temperature, sometimes a very considerable one, and it is especially noteworthy that this was always accompanied by an improvement in the patients' condition. They slept and had better appetites. When the temperature rose again, fresh injections were made, these being often administered daily for 8 or 10 consecutive days. A definitive fall of temperature after one injection occurred once.

In some cases death occurred in spite of the injection. In others the fatal termination was postponed, as in a case of pyemia with numerous pulmonary abscesses. The remedy seems to possess the power of depressing the temperature and the pulse; and it has a certain diagnostic value in addition to its good effect upon the patient. Fehling is decidedly of the opinion that collargolum should be employed in the treatment of puerperal fever, though further experimentation in the clinical field is desirable.

Dr. Hiram N. Vineberg, New York, (*American Journal of Obstetrics,* September, 1903), in a paper with a similar title says:

In a patient whom I had under observation a couple of years ago, and who was ill for a long time, I made the diagnosis of septic thrombosis of the pelvic veins on the right side. The patient was very seriously ill, had repeated severe rigors, followed by high temperature. The uterus behaved normally and involution progressed as it should. There was no pelvic exudate, but I could feel a round, hard cord along the infundibulopelvic ligament; the adnexa were apparently normal; there were no signs of peritonitis. At one time during the illness a prominent internist who saw the patient in consultation concurred in my diagnosis of pelvic phlebitis and thought he found evidences of septic endocarditis. The patient was in a precarious condition for weeks, but finally made a good recovery, the treatment consisting of the usual stimulating and nourishing agents employed to combat sepsis, together with free inunctions of unguentum Credé. There were no external metastatic abscesses. There was no bacteriologic examination made of the uterine discharge, for the reason that when the sepsis became manifest, which was rather late in the puerperium, there was practically no discharge from the uterus. I had reason to suspect a gonorrheal infection, as the husband had suffered from an acute attack of gonorrheal urethritis a short time before the wife conceived, and during the early stages of the pregnancy there was a marked erosion of the cervix with a copious mucopurulent discharge.

It may be appropriate here to say a few words in reference to the use of collargolum or unguentum Credé. I am in the habit of using unguentum Credé in cases of sepsis where I can find no lesion which demands surgical intervention, or in those cases in which the gross source of infection has been removed by surgical means and the manifestations of sepsis still persist. I have gained the impression from its use that it is of some service, either in aiding the system to eliminate the toxins produced or in some way counteracting their deleterious effects. Certain it is that several desperate cases in which the silver was employed by inunction ended in recovery, and it did seem to me that the favorable results were in a measure induced by the inunctions of the silver.

Before closing I desire to lay especial emphasis on the importance of watching very carefully every puerperal woman who shows the slightest elevation of temperature. If it be assumed that such elevation denotes sepsis, unless some other cause unmistakably accounts for it, and the proper treatment instituted at once, then, in my opinion, it will rarely occur in private practice that a case of puerperal sepsis will be encountered in which any serious surgical intervention will be needed.

The third paper is by Dr. J. Bamberger, Kissingen, Germany, (*Berliner Klinische Wochenschrift,* August 24, 1903). Among other things he says:

In the following lines an attempt will be made to elucidate the manner in which collargolum acts, without referring to its efficacy, which has been abundantly confirmed by numerous authorities. That its action is not bactericide was demonstrated by Brunner and Cohn; and Bamberger's own experiments also established this. Cohn showed how rapidly the silver, introduced intravenously, is distributed throughout the organs; a fact which had already been proved by Siebel for indigo, ink, and the like, and which Bamberger himself corroborated with frogs by subcutaneous collargolum injections.

A collargolum injection is therefore to be regarded as an introduction into the system of extremely finely divided particles of silver. That important changes in the blood occur from such an injection may be assumed; for an enormous number of leucocytes must be concerned in removing the precipitated silver from the blood. These leucocytes certainly lose their usefulness in the body economy and leave the organism laden with silver, being excreted in the feces and in other ways. To replace the missing leucocytes, the blood-forming organs must necessarily produce new ones, which then take part in combating the infection. Leucocyte formation is stimulated by the toxins; and this stimulation is further enhanced by collargolum.

The impartial observer must be absolutely convinced that collargolum does give practical results, animal experimentation to the contrary notwithstanding. That there has been no success in some cases is probably due to the fact that it was used too late to stimulate the formation of new leucocytic hosts. In animal experiments a relatively large amount of infectious material is introduced directly into the blood current; and these violent infections are not properly comparable to infections which occur in everyday life, where the germs often get into the blood late and often not at all. Under the natural conditions the body has more time to take protective measures against bacterial invasion; and a well-timed collargolum injection will help the organism powerfully, though, indeed not through any bactericide properties of the drug.

Another legitimate criticism to many animal experiments is that the collargolum was injected before the infective material. Thus the infection reached the blood at a time when a large proportion of the leucocytes was busied with the silver particles and the organism was robbed of an important element of protection. Beyer waited with his collargolum injection until the animals showed signs of violent sickness; and they got well. Animal experiments made by Bamberger showed to what an extent the leucocytes are engaged in carrying off the grains of silver injected subcutaneously.

In order to prove practically his theoretical conclusion that after a large portion of the leucocytes have left the system, stuffed full of silver, an important leucocytosis occurs, Bamberger inuncted for 30-35 minutes 45 grains of the ointment on his leg, repeating this three times. Nothing was eaten between each inunction and blood count. The following table gives his results:

| | No. of leucocytes before inunction. | 15 minutes after. | 2 hours after. | 5 hours after. |
|---|---|---|---|---|
| First inunction | 3,700 | 3,100 | 2,500 | 5,000 |
| Second inunction | 4,700 | 3,300 | 3,000 | 5,200 |
| Third inunction | 4,500 | 3,400 | 3,300 | 6,300 |

Theoretically, a direct bactericide action of the silver can only occur when abscess formation has set in. Silver-laden leucocytes would probably be found among those called forth by the inflammatory reaction, for metallic silver hinders bacterial growth in its immediate vicinity. It is possible that a part of the efficacy of collargolum may be due to its catalytic properties as a colloidal metal.

